# Bio-Inspired Microstructural Engineering of Polyurethane Foams with Luffa Fibers for Synergistic Optimization of Ergonomic Support and Hygrothermal Comfort

**DOI:** 10.3390/polym18030320

**Published:** 2026-01-25

**Authors:** Mengsi Zhang, Juan Zhou, Nuofan Tang, Yijun Hu, Fuchao Yan, Yuxia Chen, Yong Guo, Daowu Tu

**Affiliations:** 1 School of Materials & Chemistry, Anhui Agricultural University, Hefei 230036, China; 15212502135@163.com (M.Z.); zj21720331@163.com (J.Z.); 19965399553@163.com (N.T.); hu1014920@163.com (Y.H.); 19829607338@163.com (F.Y.); sheherose@ahau.edu.cn (Y.C.); 2 Anhui Healthy Sleep Home Furnishing Engineering Research Center, Hefei 230036, China

**Keywords:** polyurethane foam, luffa fiber, microstructural regulation, ergonomic pillow, thermo-hygrometric comfort

## Abstract

Traditional flexible polyurethane (PU) foams frequently exhibit limited mechanical support and suboptimal moisture–heat regulation, which can compromise the microenvironmental comfort required for high-quality sleep. In this study, natural luffa fibers (LF) were incorporated as a microstructural modifier to simultaneously enhance the mechanical and moisture–heat regulation performance of PU foams. PU/LF composite foams with varying LF loadings were prepared via in situ polymerization, and their foaming kinetics, cellular morphology evolution, and physicochemical characteristics were systematically investigated. The results indicate that LF functions both as a reinforcing skeleton and as a heterogeneous nucleation site, thereby promoting more uniform bubble formation and controlled open-cell development. At an optimal loading of 4 wt%, the composite foam developed a highly interconnected porous architecture, leading to a 7.9% increase in tensile strength and improvements of 19.4% and 22.6% in moisture absorption and moisture dissipation rates, respectively, effectively alleviating the heat–moisture accumulation typically observed in unmodified PU foams. Ergonomic pillow prototypes fabricated from the optimized composite further exhibited enhanced pressure-relief performance, as evidenced by reduced peak cervical pressure and improved uniformity of contact-area distribution in human–pillow pressure mapping, together with an increased SAG factor, indicating improved load-bearing adaptability under physiological sleep postures. Collectively, these findings elucidate the microstructural regulatory role of biomass-derived luffa fibers within porous polymer matrices and provide a robust material basis for developing high-performance, sustainable, and ergonomically optimized sleep products.

## 1. Introduction

With the growing societal emphasis on health and well-being, consumers increasingly demand higher-performance household products, particularly bedding materials, which play a critical role in sleep quality. Flexible polyurethane (PU) foam, widely used as the primary cushioning material in pillows, is valued for its excellent energy-absorbing capacity, tunable firmness, and cost-effectiveness [1,2]. However, pristine PU foams generally exhibit two major limitations that limit further advancement in comfort performance. First, their mechanical support is often inadequate; prolonged cyclic loading can readily induce permanent deformation, thereby reducing cervical support and adversely affecting musculoskeletal health and sleep quality [3]. Second, their predominantly closed-cell or semi-closed-cell microstructure is typically associated with low air permeability and limited heat/moisture transport, causing heat and moisture generated around the head during sleep to accumulate within the foam. This accumulation leads to an undesirable muggy sensation and compromises the microenvironmental comfort essential for sleep [4,5]. Therefore, developing next-generation PU foams that simultaneously enhance mechanical support and enable effective moisture–heat regulation has become an urgent research priority and a key scientific challenge in advancing high-performance sleep-comfort materials.

Functional modification of polymer matrices using natural biomass fibers has emerged as an effective approach to improve material performance while supporting sustainable development initiatives [6]. Among these bio-derived reinforcements, luffa fiber (LF) has attracted considerable interest due to its distinctive physical architecture and chemical composition, which together confer strong potential as both a reinforcing phase and a functional modifier for polyurethane (PU) foams. LF possesses an intrinsic three-dimensional, interconnected porous network, and its continuous channel structure can facilitate moisture and heat transport within the foam matrix [7,8]. Moreover, as a typical lignocellulosic material, LF contains abundant hydrophilic hydroxyl (–OH) groups on its surface, endowing it with favorable moisture-adsorption capability [9]. In addition, its inherently lightweight yet mechanically robust nature enables LF to act as a reinforcing skeletal component within the PU matrix, thereby improving the structural integrity and mechanical support of the resulting foam [10]. Accordingly, incorporating this natural fiber—combining intrinsic porosity, hydrophilicity, and high toughness—into PU foam systems represents a promising strategy to concurrently address the dual limitations of inadequate mechanical support and suboptimal thermo-hygrometric comfort in conventional foams.

In recent years, there has been growing interest in incorporating natural cellulose-based materials, such as luffa fibers, into polyurethane composite foams to enhance their overall performance. While prior research has demonstrated improvements in properties like tensile strength, hardness, and thermal stability when incorporating natural fibers into PU foams, our study goes beyond these single-property enhancements by addressing both mechanical support and thermo-hygrometric comfort through the novel incorporation of luffa fibers. This study establishes a more integrated view of foam performance, linking microstructure, mechanical properties, and comfort metrics, which is largely absent in earlier works [11,12]. However, several notable limitations remain in the current body of research. First, the research perspective is still relatively narrow, as most studies emphasize improving a single macroscopic property (e.g., mechanical strength or moisture uptake) without establishing a systematic relationship between fiber incorporation and the concomitant evolution of foam microstructure. As a result, the fundamental mechanisms responsible for performance enhancement are not yet fully clarified. Second, synergistic analyses are insufficient. Only a limited number of investigations have pursued the concurrent optimization of two core comfort-related attributes—mechanical support capacity and thermo-moisture transport behavior—which are intrinsically coupled and equally critical for practical applications. Third, a clear disconnect persists between laboratory-scale material characterization and end-use product performance. In particular, many studies do not directly link modified foam properties to ergonomic comfort indicators relevant to real products, such as pressure-distribution characteristics in pillows. Consequently, a substantial gap remains between experimental outcomes and application-driven requirements, which limits the translational relevance of existing research.

To address these gaps, this study proposes a design strategy in which luffa fibers are not treated merely as passive fillers, but rather as functional microstructural modulators.

By integrating microstructural regulation with systematic characterization of mechanical and hygrothermal transport properties and product-level human–pillow pressure mapping, this work establishes an explicit “composition–microstructure–property–ergonomic performance” linkage that is often not addressed in prior PU foam–fiber studies. The results demonstrate that the material-level improvements enabled by LF-driven porous-network engineering can be translated into measurable enhancements in end-use comfort, confirming the transfer of microstructural and mechanical advantages to practical ergonomic performance. The novelty of this study lies in the unique ‘composition–microstructure–property–ergonomic performance’ framework, which explicitly links material modifications with ergonomic outcomes, a relationship that has not been fully explored in previous research on luffa-based polyurethane foams.

## 2. Materials and Methods

### 2.1. Materials

The polyurethane foam components used in this study were provided by Viscor Sponge Co., Ltd. (Jiaxing City, Zhejiang Province, China). The polyols employed included polyether polyol 1621 (hydroxyl value: 42 mg KOH/g; moisture content: 0.06%; viscosity at 25 °C: 900 mPa·s), polyether polyol 307 (hydroxyl value: 240 mg KOH/g; moisture content: 0.10%; viscosity at 25 °C: 260 mPa·s), and polyether polyol 3630 (hydroxyl value: 24.5 mg KOH/g; moisture content: 0.05%; viscosity at 25 °C: 3000 mPa·s). The isocyanate component consisted of a mixture of diphenylmethane diisocyanate (MDI) and polymethylene polyphenyl isocyanates with an NCO content of 26.5 wt%. The catalyst system comprised triethylenediamine, bis(2-dimethylaminoethyl) ether, NIAX Catalyst A-300, and triethanolamine. A silicone-based surfactant (BA-7810) was used to stabilize the foam structure and control cell size during the foaming process. Dichloromethane and water served as foaming agents, with water functioning as a chemical blowing agent that reacts with isocyanates to produce carbon dioxide, thereby driving foam expansion. The isocyanate index of the formulation was maintained at 0.75.

Luffa fiber: The luffa material used in this study (*Luffa cylindrica* (L.) Roem.) was sourced from Lu’an City, Anhhui Province, China. The raw luffa was processed through soaking, deseeding, washing, drying, cutting, grinding, and sieving to obtain fine luffa powder for subsequent use. Pillow samples of different heights were fabricated in accordance with ergonomic standards for commonly observed anthropometric ranges, including 6, 8, 10, 12, and 14 cm for the supine posture, and 8, 10, 12, 14, and 16 cm for the lateral posture. All pillow samples were produced using the same mold to ensure geometric consistency across formulations.

### 2.2. Methods and Equipment

#### 2.2.1. Methods

**Figure 1:**
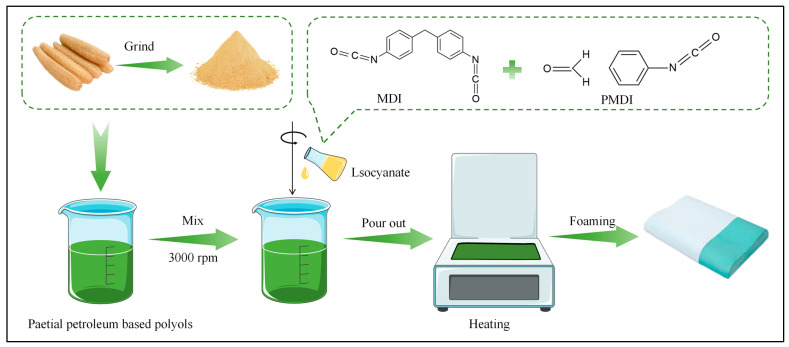
Flow chart for the preparation of luffa-modified polyurethane foam.

The luffa-modified polyurethane foams used in this study were prepared by introducing luffa fiber as a functional modifier into the polyurethane matrix, followed by foaming through a viscoelastic PU foaming process to obtain the composite foams. The specific process is shown in Figure 1. The formulations were modified by incorporating luffa fiber at loadings of 2%, 4%, 6%, and 8% relative to the total polyol mass, while the L-F0% sample served as the unmodified control without luffa addition. Prior to testing, all samples were conditioned for 24 h in a controlled environmental chamber maintained at 23 °C and 50% relative humidity. During preparation, the required amounts of polyether polyols, catalysts, blowing agents, and other constituents were accurately weighed according to the formulation and transferred into a 2000 mL polypropylene beaker. At room temperature, the mixture (Component A) was homogenized using an overhead mechanical stirrer at 3000 rpm for approximately 20 s. MDI (Component B) was subsequently added to Component A and mixed for 6–8 s to ensure rapid and uniform blending. The reactive mixture was then poured into a mold preheated to 40 °C to initiate foaming. After foaming, specimens were demolded after 10 min and conditioned at room temperature for 72 h before dimensional trimming and subsequent physicomechanical characterization. To justify the selected luffa range, the formulations were designed from 0 to 8 wt% (0, 2, 4, 6, and 8 wt%) to cover a practically processable window while capturing the transition from cell refinement at moderate loading to viscosity-driven foaming instability at higher loading.

All results were subjected to statistical analysis using one-way analysis of variance (ANOVA) to compare the means of the different formulations. Post hoc Tukey’s test was applied to assess differences between individual groups when ANOVA indicated a statistically significant effect. *p* < 0.05 was considered statistically significant. However, in some cases, the differences were not statistically significant (*p* > 0.05). Despite the lack of statistical significance, the observed trends and variations were considered informative and are discussed in the manuscript. The error bars in the figures represent the standard deviations of the measurements.

#### 2.2.2. Equipment

The microstructural morphology of the foams was characterized using a scanning electron microscope (SEM, S-4800, Hitachi, Japan). Observations were performed at an accelerating voltage of 3 kV, and suitable magnifications were selected to evaluate the cellular architecture and pore-size distribution.

The characteristic parameters of the foaming reaction were determined by initiating a stopwatch immediately upon mixing Components A and B. The cream time was recorded at the onset of bubble formation accompanied by visible whitening of the mixture. The gel time was identified when a thin filament could be drawn from the surface of the reacting mixture using a glass rod. The tack-free time was defined as the moment when the foam surface no longer exhibited adhesion upon gentle contact with the glass rod. For consistency, all reaction times were recorded in seconds (s).

Fourier transform infrared (FTIR) spectroscopy was used to characterize the PU foams with varying luffa fiber loadings. The spectra were recorded over a wavenumber range of 400–4000 cm^−1^.

In accordance with ISO 2439:2008(E) [13], hysteresis loss, compression deflection coefficient, and indentation hardness were measured using a universal testing machine (SHIMADZU AG-X Plus, Kyoto, Japan) on luffa-modified PU foam specimens with dimensions of 20 × 20 × 10 cm^3^. For the determination of hysteresis loss (Af), the specimens were compressed to (75 ± 1)% of their original thickness and subsequently unloaded. Tensile strength, elongation at break, and tear strength were evaluated following the corresponding mechanical testing procedures. The tensile properties of the luffa-modified PU foams were further assessed according to ISO 1798:2008 [14] using Type 1A dumbbell-shaped specimens, which were stretched at a crosshead speed of 500 mm/min during testing.

For the determination of apparent density and porosity, the foams prepared in the previous section were cut into cubic specimens (10 mm × 10 mm × 10 mm) using a foam-cutting apparatus, with five replicates prepared for each formulation. The specimens were conditioned for 24 h in a controlled environmental chamber maintained at 24 °C and 65% relative humidity (HWS-P250C), after which they were removed and weighed using an analytical balance (FA2004, Beijing, China; accuracy 0.1 mg) to obtain the mass m. The length, width, and height of each specimen were measured using a stainless-steel caliper (Shanghai, China; accuracy 0.01 mm), and the specimen volume v was subsequently calculated. The apparent density of each specimen was then determined using Equation (1).

(1) $$\rho =m/v\;*\;10^{6}$$

Porosity was determined using the ethanol displacement method. The initial volume of ethanol was recorded as V_1_. The foam specimen was then immersed in ethanol, and the resulting volume was recorded as V_2_. After immersion, the specimen was removed, and the ethanol volume was measured again and recorded as V_3_. Based on these measured values, the porosity was calculated according to Equation (2).

(2) $$P=V_{1}-V_{2}/V_{2}-V_{3}$$

The moisture absorption behavior of the foams was evaluated using a gravimetric method in accordance with ASTM D5229 [15].

The moisture desorption performance of the foams was evaluated using a gravimetric method in accordance with ISO 22649:2016 [16].

Body pressure distribution was evaluated using a Body Pressure Measurement System (BPMS; Tekscan, South Boston, MA, USA), consisting of a pressure-sensing mat and a handheld interface. Measurements were recorded for 3 min at a sampling frequency of 8 frames·s^−1^, and the interface pressure was documented in units of kPa. Specifically, our research subjects included 16 healthy college student volunteers without any musculoskeletal pain symptoms. Among them, there were 8 males, aged 24 ± 2 years, with a height of 175.4 ± 10 cm and a weight of 66.2 ± 5.3 kg; and 8 females, aged 24.1 ± 1 year, with a height of 161 ± 3.9 cm and a weight of 54.5 ± 4.8 kg. The subjects covered a wide range of body types (including individuals of relatively small, medium, and large body sizes for both males and females), in order to enhance the applicability of the body pressure test results to people of different body types. Regarding repeatability, we ensure that each test is conducted on the same object multiple times to minimize the differences in the experiment. Specifically, each test is repeated three times to assess the consistency of the results.

#### 2.2.3. Moisture Transfer Model

In order to simulate the water transfer process in the modified cucumber polyurethane foam, we adopted a theoretical framework based on the mature diffusion principle. This model takes into account the hygroscopic and dehygroscopic behaviors of the foam under different conditions. To enhance the clarity and reproducibility of the water transfer simulation model, we clearly defined the key assumptions behind the model. Firstly, we regarded the modified cucumber polyurethane foam as a continuous porous medium, which simplified the model and represented the foam’s cell structure as a uniform material, thus avoiding the complexity of the interactions between individual cells. Secondly, the model is based on the local equilibrium assumption, which states that the water content at any point in the foam at each time step is in equilibrium with the surrounding environment. This assumption is crucial for the application of standard water diffusion models and enables us to describe the water transfer process in a computationally efficient manner.

Secondly, establishing the water absorption and release model involves constructing a theoretical framework, fluid flow model, mass transfer model, and coupled water absorption–release model.

As for the analysis of water absorption and release in the pillow-like structure, the model development includes geometric construction, definition of relevant physical fields, mesh generation, solver configuration, and numerical calculation.

## 3. Results and Discussion

### 3.1. Scanning Electron Microscopy (SEM) Analysis

**Figure 2:**
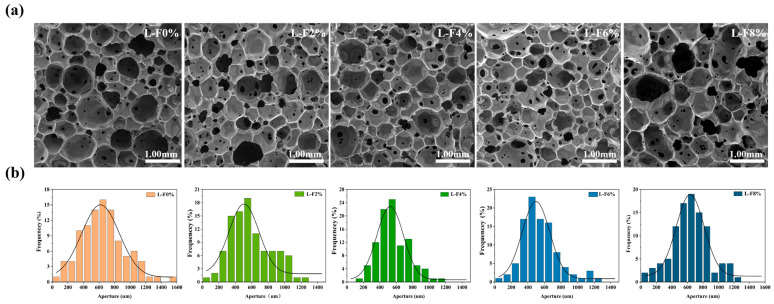
(**a**) Scanning electron micrographs. (**b**) Vesicle diameter distribution.

SEM observations revealed pronounced alterations in the cellular morphology of the PU foams with increasing luffa fiber content. As shown in Figure 2a,b, increasing fiber loading initially resulted in a reduction in cell diameter together with a gradual increase in the number of open cells; however, further fiber addition caused an increase in cell diameter accompanied by a decrease in open-cell count. Table S1 (Supplementary Materials) lists the pore diameters of the luffa-fiber-modified polyurethane foams. Specifically, most cells in the L-F0% foam exhibited diameters in the range of 500–700 μm, whereas the cell-size distributions for L-F2%, L-F4%, L-F6%, and L-F8% shifted to 450–650 μm, 400–600 μm, 450–650 μm, and 500–700 μm, respectively. The average cell diameter followed a characteristic decrease–increase trend with rising luffa content, with L-F0% displaying the largest mean size and L-F6% the smallest. This phenomenon can be attributed to the elevated viscosity of the foaming system induced by fiber incorporation, which restricts bubble expansion during the early stage of foaming. Meanwhile, the fibers introduce a greater number of heterogeneous nucleation sites, thereby promoting cell initiation and refinement [17]. By lowering the nucleation energy barrier, luffa fibers facilitate bubble formation and contribute to the development of finer cellular structures. Accordingly, at fiber loadings of 0–4%, the foams exhibited smaller average cell diameters and more uniform cellular morphologies.

When the luffa fiber content exceeded 4%, particularly at loadings of 6% and 8%, the viscosity of the reaction system increased markedly, which impeded normal cell-wall expansion and promoted wall rupture and/or cell coalescence, ultimately leading to irregular enlarged cells and localized collapse phenomena [18]. This structural transition is clearly evident from the SEM micrographs: in the L-F6% and L-F8% foams, the originally well-defined hexagonal cells were distinctly distorted into irregular polygonal morphologies, accompanied by a more heterogeneous cell-size distribution. These observations suggest that excessive luffa incorporation induces progressive fiber agglomeration, and the resulting aggregates act as stress concentrators that trigger cell-wall rupture, thereby generating irregular and non-uniform cellular architectures. This trend is consistent with the findings of Charles Kuranchie et al., who reported analogous structural degradation in polyurethane foams modified with clove powder and neem oil, where excessive additive content led to cell-wall rupture, cavity formation, and strut damage attributed to partial collapse during foam rise [19]. Their SEM observations further indicated particle accumulation along foam struts, disrupting the continuity and uniformity of the cellular network [20]. In line with these reports, the present study demonstrates that lower luffa loadings (0–4%) yield foams with more uniform cell structures due to effective fiber dispersion within the matrix, whereas higher loadings (6–8%) severely compromise dispersion, promote cell-wall rupture, and induce pronounced distortion of the cellular morphology.

Compared with the unmodified foam (L-F0%), the luffa-modified PU foams exhibited a markedly higher proportion of open-cell structures. With increasing luffa content, the number of open cells increased progressively, and the most pronounced enhancement was observed for the L-F2% to L-F4% formulations. However, further increases in luffa loading (L-F6% and above) resulted in a reduced open-cell count. These results indicate that moderate luffa incorporation facilitates the optimization of cellular architecture and improves cell uniformity, whereas excessive fiber addition drives the formation of an overly open and structurally less stable cell network, thereby compromising foam stability and diminishing mechanical performance.

### 3.2. Physicochemical Property Analysis

Incorporating additives, particularly fillers, into polyurethane foam formulations not only enhances the performance of the resulting PU composite materials but also modifies the kinetics of foam formation and can shift the chemical balance of the foaming reactions [21,22]. The cream time, gel time, and tack-free time are governed by multiple factors, including the chemical nature of the polyols, the ratio of primary to secondary hydroxyl groups in the reaction mixture, interactions between the hydroxyl groups of the additives and the polyols, and the amount of water introduced into the formulation [23,24]. In general, the cream time of polyurethane foams is defined as the interval between the onset of emulsification and the initiation of foam rise, thereby marking the beginning of the gelation process, during which the viscosity of the reacting mixture progressively increases [25]. The gel time corresponds to the stage at which emulsification ceases and foam rise begins, and thus reflects the progression and rate of the foaming reaction. Under appropriate formulation conditions, the foam rises gradually and continuously, ultimately expanding several centimeters above the mold before the rise process terminates, after which the foam is allowed to cure briefly and a thin, tacky surface “skin” forms [26]. Accordingly, the tack-free time denotes the point at which the foam stops rising and the surface is no longer tacky; at this stage, the foam can be demolded and subsequently cured to develop a well-formed polymer network [27]. Based on these definitions, the cream time, rise (expansion) time, and gel time of luffa-modified polyurethane foams with different fiber loadings were systematically monitored.

**Figure 3:**
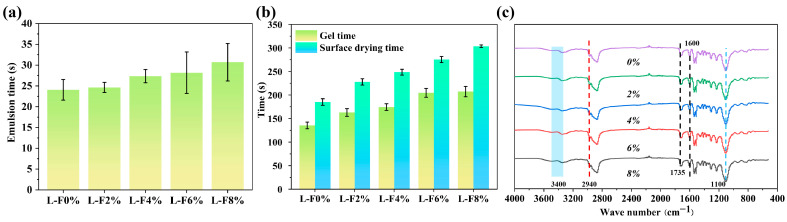
(**a**) Emulsion time. (**b**) Gel and surface drying time of luffa-modified polyurethane foam. (**c**) FTIR plots of luffa-modified polyurethane foam.

As the luffa fiber content increased, the cream time, gel time, and tack-free time of the luffa-modified polyurethane foams increased progressively, as shown in Figure 3a,b, and Table S2 (Supplementary Materials). The L-F8% formulation exhibited the longest cream, gel, and tack-free times, indicating the most pronounced retardation of foaming kinetics. The prolonged cream time may be attributed to the additional resistance to bubble expansion introduced by fiber incorporation, which slightly delays the onset of foam rise and the early-stage foaming reaction [28]. A similar tendency was reported by Zhang et al., who found that both cream time and gel time increased with higher loadings of straw-based fillers [28]. Ribeiro da Silva et al. observed that incorporating rice husk ash at concentrations of 2 wt% to 5 wt% increased the cream time from 15 s to 22 s [29]. Likewise, Zukowska et al. reported that increasing the content of recycled tire rubber particles from 5 wt% to 20 wt% in polyurethane foam formulations resulted in a pronounced extension of tack-free time [30]. In addition, the increased viscosity of the reacting mixture at higher fiber contents can hinder homogeneous dispersion of additives within the polyurethane matrix, thereby delaying the onset of both gelation and the tack-free stage of the foaming process [29].

Figure 3c shows the FTIR spectra of polyurethane foams containing different luffa fiber loadings. As shown in Figure 3c, the spectra of L-F2%, L-F4%, L-F6%, and L-F8% are highly similar to those of the unmodified foam (L-F0%), indicating that luffa incorporation does not substantially alter the main chemical structure of the PU matrix within the detectable range. The broad band at 3200–3400 cm^−1^ is assigned to the symmetric and asymmetric stretching vibrations of N–H (amide/urethane) groups [20]. The bands in the range of 2820–2980 cm^−1^ are mainly attributed to the stretching vibrations of –CH_2_ groups associated with the polyol soft segments [31]. A characteristic peak at 2940 cm^−1^ is assigned to –CH_2_ stretching in aliphatic carbon chains [32], whereas the band at 1735 cm^−1^ is attributed to the C=O stretching vibration of the Amide I band [20]. The absorption bands at 1600 and 823 cm^−1^ are assigned to aromatic ring vibrations inherent to the foam formulation [20]. The peaks in the 1540–1519 cm^−1^ region, together with the band at 1450 cm^−1^, are attributed to the Amide II band, arising primarily from N–H bending and C–N stretching vibrations associated with urethane/urea C–N–H groups [33]. In addition, the absorption region at 1410–1350 cm^−1^ is mainly related to the bending and wagging vibrations of methylene (C–H) groups [20]. Another distinct peak at 1305 cm^−1^ corresponds to the C–N stretching vibration of urethane groups [20]. In addition, the absorption at 1225 cm^−1^ is assigned to the asymmetric stretching of C–O–C (ether) in the Amide III band, together with contributions from N–H bending and C–N stretching [20]. A peak at 1100 cm^−1^ is associated with C–O stretching vibrations of C–O–C groups, including contributions from carbamate and ether linkages [20]. Compared with the unmodified sample, slight shifts in several absorption bands were observed for the luffa-modified foams, suggesting that fiber incorporation during foaming promoted hydrogen-bond formation within the composite system. Specifically, the abundant hydroxyl (–OH) groups on luffa fibers can establish hydrogen-bond interactions with the carbonyl (C=O) groups in the polyurethane hard segments, thereby strengthening interfacial adhesion between the fibers and the PU matrix and contributing to the enhanced mechanical performance of the composite foams. Moreover, these interfacial interactions provide additional reinforcement to the matrix, which is consistent with the observed improvements in both tensile strength and tear resistance.

### 3.3. Mechanical Performance Analysis

**Figure 4:**
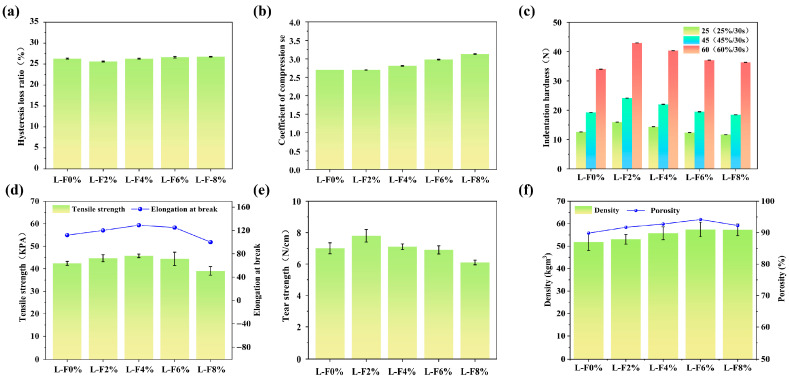
(**a**) Hysteresis loss ratio, (**b**) coefficient of compression set, (**c**) indentation hardness, (**d**) Tensile strength, elongation at break, (**e**) tear strength, (**f**) density and porosity.

As shown in Figure 4a and Table S3 (Supplementary Materials), the hysteresis loss of the foams exhibited an initial decrease followed by an increase with rising luffa content, decreasing from 26.3% to 25.6% and then increasing to 26.76%. The L-F2% sample showed a lower hysteresis loss than the unmodified foam, whereas the other formulations exhibited higher values, with L-F8% presenting the highest hysteresis loss. Figure 4b shows the compression deflection coefficient of the foams. As shown in Figure 4b, the coefficient of L-F2% was comparable to that of L-F0%, while the coefficients of the other formulations increased progressively with increasing luffa content. The compression deflection coefficient, also termed the comfort factor, is a key parameter for evaluating the comfort-related performance of foam materials [34], and values of 1.8 or higher are generally considered indicative of adequate comfort. According to Figure 4b and Table S3 (Supplementary Materials), all luffa-modified polyurethane foams exhibited compression deflection coefficients above 1.8. Notably, both L-F0% and L-F2% presented relatively low coefficients (2.70), which not only satisfied the minimum comfort criterion but also exceeded the conventional threshold associated with satisfactory comfort. Overall, these results indicate that the luffa-modified polyurethane foams possess favorable compression deflection characteristics, highlighting their potential for improved comfort performance.

Foam indentation hardness is a key parameter for evaluating the softness and comfort-related performance of polyurethane foams. As shown in Figure 4c and Table S3 (Supplementary Materials), the indentation hardness of the luffa-modified foams exhibited a distinct trend of first increasing and then decreasing with increasing luffa content under the applied loads. The L-F2% sample displayed the highest indentation hardness, followed by L-F4%. Both L-F2% and L-F4% showed higher indentation hardness values than L-F0%, whereas L-F6% and L-F8% exhibited values that were comparable to those of the unmodified foam. These results suggest that luffa incorporation at appropriate loadings can beneficially modulate the indentation response, thereby supporting improved softness and comfort characteristics of the PU foam.

For polyurethane foams, tensile strength reflects the material’s resistance to tensile deformation under applied load [35]. Foams with higher tensile strength are better able to withstand tensile forces, thereby offering improved resistance to tensile loading and making them suitable for tensile-load applications [36]. The elongation at break reflects the deformation capacity of polyurethane foams under tensile loading. In general, higher elongation at break indicates improved ductility, allowing the material to absorb energy and mitigate stress concentrations within the loaded region, thereby reducing damage associated with external impact or deformation [37]. As shown in Figure 4d and Table S4 (Supplementary Materials), the bar chart represents the tensile strength of the luffa-modified foams, while the line plot depicts the corresponding elongation at break. Both parameters exhibited a trend of initially increasing and subsequently decreasing with increasing luffa content. Specifically, the tensile strength increased from 42.39 kPa to a maximum of 45.73 kPa and then decreased to 39.05 kPa. Similarly, as shown in Figure 4e, the elongation at break increased from 112% to 129% before decreasing to 100%. Among all formulations, L-F4% achieved the highest tensile strength and elongation at break, corresponding to increases of 7.9% and 15.2%, respectively, relative to L-F0%. In contrast, L-F8% exhibited the lowest tensile strength and elongation at break, both of which were inferior to those of the unmodified foam, whereas the remaining formulations showed improved values compared with L-F0%. These results indicate that an appropriate luffa loading enhances both tensile performance and ductility, which is beneficial for durability during service. The observed increase–decrease pattern suggests that at lower contents (L-F2% and L-F4%), luffa fibers are more effectively dispersed and act as reinforcement within the matrix, thereby improving strength and ductility. However, when the luffa content exceeds 4% (L-F6% and L-F8%), fiber agglomeration becomes more pronounced, generating stress-concentration sites that facilitate premature failure of the foam matrix and consequently reduce both tensile strength and elongation at break [38]. Mechanistically, at low fiber loadings (2–4 wt%), the refined and more uniform cellular architecture (smaller cells with reduced geometric defects) promotes a more homogeneous stress distribution within the strut–cell-wall network, delaying local yielding and crack initiation under tensile loading. In parallel, well-dispersed luffa fibers can act as micro-bridges across adjacent struts/cell walls, enabling more effective load transfer from the PU matrix to the fiber skeleton; this synergizes with the enhanced interfacial adhesion (as evidenced by the FTIR-indicated hydrogen-bonding interactions) to suppress interfacial debonding and improve ductility. By contrast, at higher loadings (≥6 wt%), fiber agglomerates disrupt cell growth and introduce irregular large cells and weakened struts, thereby amplifying stress concentration and facilitating early cell-wall rupture; once cracks nucleate at these defect-rich regions, rapid propagation through the compromised cellular network results in premature failure and reduced elongation.

The tear resistance of polyurethane foams is an important indicator of long-term stability and service reliability. Foams with higher tear strength can better resist externally applied tearing forces, thereby reducing the risk of tear-initiated failure and enhancing durability in practical applications [39]. As shown in Figure 4e and Table S4 (Supplementary Materials), the tear strength exhibited a characteristic increase–decrease trend, rising from 7.0 kPa to 7.8 kPa and then declining to 6.1 kPa. Among the formulations, L-F2% achieved the highest tear strength, representing an 11.4% increase relative to L-F0%. The tear strengths of L-F2% and L-F4% remained higher than that of the unmodified foam, whereas those of L-F6% and L-F8% were lower. Overall, these results suggest that luffa incorporation at an appropriate loading can improve tear resistance, thereby supporting enhanced durability and service reliability of polyurethane foams.

### 3.4. Moisture Absorption and Desorption Performance Analysis

The density and porosity of polyurethane foams are fundamental parameters for characterizing their cellular architecture. These structural features are closely associated with moisture absorption and desorption behavior, which is closely linked to the material’s moisture absorption and desorption behavior. As shown in Figure 4f and Table S5 (Supplementary Materials), the apparent density and porosity of the foams containing different luffa loadings differed noticeably among formulations. With increasing luffa content, both apparent density and porosity exhibited an initial increase followed by a gradual decrease, reaching their maximum values at a luffa loading of 6%. In addition, the apparent density and porosity of all luffa-modified foams were higher than those of the unmodified sample. Consistent with the SEM observations in Figure 2a, the luffa-modified foams exhibited a higher open-cell population than the pristine foam. The progressive increase in open-cell content with increasing luffa loading correspondingly contributed to the increase in porosity. Enhanced cellular openness not only leads to a larger overall foam volume but also provides additional pathways for moisture transport. This structural change is therefore beneficial for moisture uptake and release in the polyurethane foams [40].

**Figure 5:**
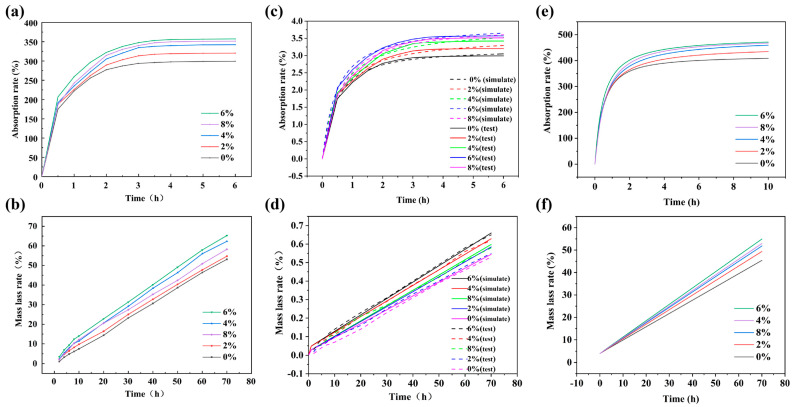
(**a**) Moisture absorption properties of luffa-modified polyurethane foam. (**b**) Dehumidifying property of luffa modified polyurethane foam. (**c**) Moisture absorption properties of luffa-modified polyurethane foam. (**d**) Validation of moisture dispersion modeling of luffa-modified polyurethane foam. (**e**) Moisture-absorbing properties of luffa-modified polyurethane foam sleeping pillows. (**f**) Moisture-dissipating properties of luffa-modified polyurethane foam sleeping pillows.

As shown in Figure 5a, the incorporation of luffa fibers effectively improved the moisture-absorption performance of the polyurethane foams, with all luffa-modified samples exhibiting higher absorption rates than the unmodified foam. Among the formulations, L-F6% showed the highest absorption capacity, with the 6 h moisture-absorption rate increasing by 19.4% relative to the unmodified sample. This was followed by L-F8% and L-F4%, for which the 6 h absorption rates were elevated by 17.4% and 14.4%, respectively, compared with L-F0%. The differences in moisture-absorption behavior among the luffa-modified foams are closely related to variations in pore structure. The porosity values were largely consistent with the moisture-absorption results, with L-F6% exhibiting both the highest porosity and the strongest moisture-absorption capability. Although slight deviations were observed between the porosity trend and the moisture-absorption behavior of L-F8% and L-F4%, these discrepancies were minor. By contrast, L-F0%, L-F2%, and L-F4% showed fully consistent trends in both porosity and moisture-absorption performance. Because porosity directly influences moisture-uptake capacity, higher porosity provides additional internal space for luffa-modified polyurethane foams to accommodate absorbed moisture. During sleep, the human body continuously releases moisture through perspiration. If this moisture is not effectively absorbed, it may accumulate at the skin–bedding interface and increase perceived dampness and stickiness. Such accumulation can also elevate local thermal discomfort and compromise the dryness of the micro-sleep environment, thereby reducing overall comfort.

As shown in Figure 5b, luffa fiber incorporation markedly improved the moisture-desorption performance of the polyurethane foams, with all luffa-modified samples exhibiting higher desorption rates than the unmodified foam. Among the formulations, L-F6% displayed the highest desorption capacity, with its 70 h desorption value increasing by 22.6% relative to the blank sample, followed by L-F4%, which showed a 17% increase compared with L-F0%. The variation in moisture desorption with increasing luffa content closely mirrored the trend in porosity: both parameters increased progressively, reached a maximum at L-F6%, and then decreased at higher loadings. The governing factors for moisture desorption were consistent with those affecting moisture absorption. The enhanced desorption is mainly attributed to the enriched internal pore architecture, which provides additional pathways for moisture release. As discussed above, the increased open-cell population observed in the SEM images reduces moisture-transport tortuosity and facilitates more rapid moisture release via capillary-driven mechanisms. Within the foam network, a higher open-cell content lowers resistance to vapor migration, enabling faster transport of water molecules and thereby improving desorption efficiency [41]. During sleep, the human body releases substantial moisture through perspiration and respiration, a considerable fraction of which is absorbed by pillows and mattresses. Retained moisture can reduce perceived comfort by increasing clamminess and limiting heat dissipation. It can also create a favorable environment for microbial growth, which may raise hygiene-related concerns [42]. Beyond improving comfort and reducing hygiene-related risks, favorable moisture-absorption and desorption performance also contributes to extending the service life of polyurethane foams [43]. Effective moisture regulation can alleviate humidity-induced degradation, thereby preserving structural stability and supporting long-term durability. This reduces the frequency of product replacement and provides economic benefits. It also supports environmentally responsible use by reducing polyurethane waste generation [44]. Therefore, employing luffa-modified polyurethane foam as a pillow material can effectively improve user comfort, extend product service life, and help reduce polyurethane waste.

As shown in Figure 5c,d, the simulated moisture absorption and desorption curves were in close agreement with the corresponding experimental results for polyurethane foams with different luffa loadings. Table S6 (Supplementary Materials) summarizes the root-mean-square error (RMSE) and Pearson correlation coefficients between the measured and simulated moisture-absorption datasets. RMSE reflects the deviation between model predictions and experimental measurements, and the obtained values (0.044–0.075) were consistently low, indicating only minor discrepancies and supporting the predictive accuracy of the model. The Pearson correlation coefficients, which quantify the linear association between the simulated and experimental datasets, were all close to unity (0.997–0.999), confirming an excellent linear correspondence. Likewise, Table S7 (Supplementary Materials) reports the RMSE and Pearson correlation coefficients for moisture desorption. The desorption RMSE values (0.044–0.075) again indicated minimal deviation between simulation and experiment, while the Pearson correlation coefficients (0.997–0.999) further verified strong linear consistency. Collectively, these results demonstrate that the simulation model can reliably reproduce the experimentally observed moisture absorption and desorption behaviors of the luffa-modified polyurethane foams, showing high agreement with the measured data.

As shown in Figure 5e,f, the moisture absorption and desorption curves predicted by the moisture-transfer model describe the hygrothermal behavior of luffa-modified polyurethane foam pillows with different luffa contents (0%, 2%, 4%, 6%, and 8%) over time intervals from 0 to 10 h. The incorporation of luffa fibers enhanced both moisture absorption and moisture desorption, with all luffa-modified samples exhibiting higher moisture uptake and release than the unmodified foam. During moisture absorption, the moisture content gradually approached a stable plateau, indicating that the luffa-modified pillows can absorb moisture progressively and maintain a stable absorption capacity over time. This behavior is beneficial for improving comfort under humid conditions by alleviating dampness-related discomfort, while also helping to inhibit microbial proliferation and maintain hygienic use conditions. Comparison among formulations showed that moisture-absorption performance was most favorable at luffa loadings between 4% and 8%, with the 6% formulation exhibiting the highest 10 h moisture uptake (15.4% higher than L-F0%), followed by the 8% (14.4%) and 4% (12.4%) formulations. Although the absorption capacity continued to increase at 6% and 8% loadings, the incremental improvement beyond 4% was relatively modest, suggesting that luffa contents within this range are appropriate for enhancing moisture-absorption performance. Regarding moisture desorption, all luffa-modified pillows displayed higher desorption rates than the unmodified sample, demonstrating that luffa fibers substantially improved moisture-release behavior. The desorbed moisture content increased progressively with time, reflecting the ability of the pillows to continuously release accumulated moisture and maintain a sustained desorption effect, which is important for reducing moisture-retention-related discomfort and supporting hygienic use conditions. Among the tested formulations, the 6% sample showed the highest 70 h desorption rate (21.0% above L-F0%), followed by the 4% sample (16.6% above L-F0%). Overall, the luffa-modified polyurethane foam pillows exhibited favorable moisture-absorption and moisture-desorption performance, contributing to a drier and more comfortable sleeping microenvironment while suppressing mold and bacterial growth. Furthermore, the finite element simulations used to model moisture-transfer processes provide dynamic insight into the moisture-regulation behavior of the pillows, offering guidance for optimizing pillow structure and material design.

### 3.5. Body Pressure Distribution Analysis

#### 3.5.1. Influence of Luffa Fiber Content on the Pressure-Relief Performance of the Pillow

**Figure 6:**
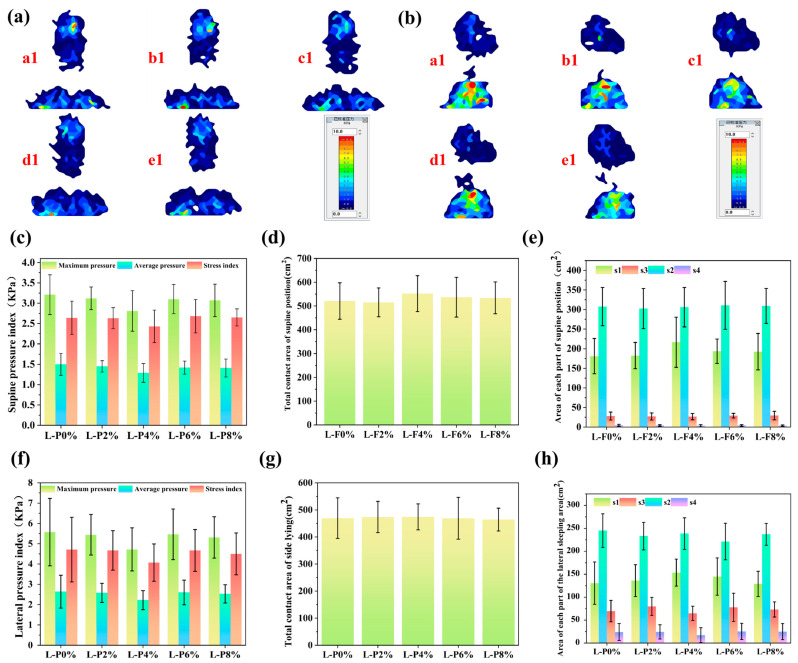
(**a**) Body pressure distribution at the human–occipital interface for (**a1**) L-P0%, (**b1**) L-P2%, (**c1**) L-P4%, (**d1**) L-P6%, and (**e1**) L-P8% (supine position). (**b**) Body pressure distribution at the human–occipital interface for (**a1**) L-P0%, (**b1**) L-P2%, (**c1**) L-P4%, (**d1**) L-P6%, and (**e1**) L-P8% (lateral position). (**c**) Pressure index of luffa-modified polyurethane foam sleeping pillows (supine position). (**d**) Sleeping pillow contact areas in supine position: total contact area. (**e**) Sleeping pillow contact areas in supine position: s1: Contact area within the pressure range below 0.67 kPa, s2: Contact area within the pressure range of 0.67–4 kPa, s3: Contact area within the pressure range of 4–9.33 kPa, and s4: Contact area within the pressure range above 9.33 kPa. (**f**) Pressure index of sleeping pillows of luffa-modified polyurethane foam (lateral position). (**g**) Sleeping pillow contact areas in lateral position: total contact area. (**h**) Sleeping pillow contact areas in lateral position: s1: Contact area within the pressure range below 0.67 kPa, s2: Contact area within the pressure range of 0.67–4 kPa, s3: Contact area within the pressure range of 4–9.33 kPa, and s4: Contact area within the pressure range above 9.33 kPa.

Figure 6a presents the head–pillow interface pressure distribution for the same subject in the supine position when using polyurethane foam pillows with different luffa fiber loadings. As shown, the L-P0% pillow exhibited a pronounced stress concentration in the head region. By contrast, the luffa-modified pillows produced a noticeably more uniform pressure distribution, with substantially reduced pressures in both the head and shoulder regions. These results indicate that incorporating luffa fibers provides an evident pressure-relief effect at the head–pillow interface. Further comparison among formulations showed that L-P4% and L-P8% delivered better pressure-relief performance than L-P2% and L-P6%, for which clear stress concentrations remained in the shoulder region. Overall, L-P4% exhibited the most favorable pressure-relief behavior. From an ergonomic standpoint, prolonged exposure of the head and neck to inappropriate pressure levels may induce excessive cervical flexion or extension, impairing local blood circulation, accelerating muscle fatigue, and potentially contributing to nerve-related discomfort or injury [45]. The luffa-modified polyurethane foam pillow exhibited the most effective pressure-relief performance at a luffa content of 4%, providing the greatest improvement in comfort under the supine posture by achieving an optimal balance between limiting excessive cervical flexion and supplying adequate compliant support.

Pressure-related parameters are essential indicators for assessing pillow comfort. As shown in Figure 6c and Table S8 (Supplementary Materials), the pressure metrics of the luffa-modified polyurethane foam pillows were evaluated under the supine sleeping posture to characterize load distribution at the human–pillow interface. The maximum pressure represents the highest localized load at a specific contact point, whereas the average pressure reflects the mean load distributed over the entire interface, and the pressure index is a composite metric commonly used to quantify pressure-distribution uniformity [46]. Previous studies have reported that the capillary closure pressure associated with skin microcirculation is approximately 32 mmHg (≈4.26 kPa); sustained interface pressures above this threshold can markedly reduce local perfusion and, over time, increase the risk of ischemic injury and pressure-related lesions [47]. In the present study, both maximum and average interface pressures exhibited a decrease–increase trend with increasing luffa content, with the L-P4% group showing the most pronounced improvement: the maximum pressure decreased from 3.21 kPa to 2.81 kPa and the average pressure decreased from 1.50 kPa to 1.29 kPa. Importantly, the maximum interface pressure for all pillow samples remained below the 4.26 kPa capillary-closure threshold, indicating a low likelihood of pressure-induced microcirculatory impairment. Within this safe range, the luffa-modified PU pillows not only reduced peak loads applied to the head and cervical region but also maintained interface pressures at levels conducive to preserving microcirculatory perfusion and metabolic homeostasis during sleep, thereby supporting both safety and overall comfort. However, when the luffa content increased to 6% and 8%, neither maximum nor average pressure decreased further, suggesting diminishing cushioning benefits beyond the optimal loading. A similar tendency was observed for the pressure index, which decreased from 2.64 kPa to 2.43 kPa and then increased to 2.65 kPa. The pressure indices of the L-P2% and L-P4% groups were lower than that of L-P0%, indicating that moderate luffa incorporation improves pressure-distribution uniformity. Overall, the L-P4% sample exhibited the lowest maximum pressure, average pressure, and pressure index, demonstrating the most effective pressure-relief performance and the strongest ability to redistribute interface loads to enhance sleep comfort.

The total contact area reflects the overall extent of interaction between the pillow surface and the human body, and a larger contact area generally indicates more uniform load redistribution at the interface, which is commonly associated with improved perceived comfort. Within the low and lower pressure-threshold ranges, a larger head–pillow contact area facilitates more effective pressure redistribution; conversely, within higher pressure ranges, a smaller contact area is preferred, as it indicates that regions subjected to elevated pressure are reduced. Figure 6d,e, and Table S9 (Supplementary Materials) present the contact-area distributions under the supine posture for pillows with different luffa fiber loadings. Specifically, the total head–pillow contact area and the contact areas within the defined pressure-threshold zones—s1 (low threshold), s2 (lower threshold), s3 (higher threshold), and s4 (high threshold)—are reported. Compared with L-P0%, the total contact area of the L-P4% pillow increased from 520.88 cm^2^ to 551.85 cm^2^, corresponding to a 5.95% increase. In the pressure range below 0.67 kPa, the L-P4% pillow exhibited the largest contact area in the supine position. Across the 0.67–4 kPa range, the contact areas of the L-P0% to L-P8% samples were broadly comparable. In contrast, within the higher pressure range of 4–9.33 kPa and the high-pressure range above 9.33 kPa, the L-P4% pillow presented the smallest contact area. Collectively, these results demonstrate the superior pressure-relief capability of the L-P4% formulation. This improvement can be attributed to the balanced mechanical response achieved at a 4% luffa loading, which provides sufficient cervical support to limit excessive flexion while maintaining adequate compliance for contouring. Such a balance increases the overall contact area, redistributes localized pressure peaks, and ultimately enhances user comfort [48].

As illustrated in Figure 6b, the body–pillow pressure distribution in the lateral sleeping posture highlights the influence of different luffa fiber contents. The pressure maps were obtained from the same participant lying laterally on pillows containing different luffa loadings, enabling a direct comparison of side-lying pressure patterns across all polyurethane foam samples. Under the lateral posture, the unmodified PU pillow (L-P0%) exhibited pronounced red regions, indicating substantial stress concentration. By contrast, all luffa-modified pillows showed noticeable improvements in pressure redistribution relative to L-P0%. Among them, the L-P4% pillow produced the most uniform body–pillow pressure distribution, suggesting superior attenuation of localized loading under lateral support conditions. During sleep, prolonged loading on the head and cervical region may cause excessive stretching or compression of cervical muscles and ligaments, potentially resulting in discomfort and, in severe cases, contributing to cervical spine disorders. Sustained compression may also impair local blood circulation, reducing perfusion and inducing symptoms such as dizziness or headache. In addition, persistent pressure in the cervical region can impose mechanical loading on peripheral nerves, leading to numbness or pain in the upper limbs and, in severe cases, increasing the risk of neurological injury. Such prolonged loading can further trigger frequent arousals, thereby disrupting sleep continuity and depth and ultimately compromising overall sleep quality [49]. Therefore, selecting a pillow with effective pressure-relief capability is essential for maintaining appropriate cervical support during sleep. With increasing luffa content, the deep-red high-pressure regions in the lateral pressure maps exhibited a characteristic trend of decreasing initially, then increasing, and subsequently decreasing again, with the smallest high-pressure area observed for the L-P4% condition. These results indicate that the luffa-modified PU pillow achieves its optimal pressure-relief performance at a luffa loading of 4%, providing the greatest improvement in lateral-sleeping comfort at this formulation level.

To evaluate the comfort performance of the luffa-modified polyurethane pillow, the pressure distribution under the lateral sleeping posture was systematically analyzed. As shown in Figure 6f and Table S10 (Supplementary Materials), the L-P0% pillow exhibited the highest maximum pressure, average pressure, and pressure index, indicating that conventional PU foam tends to generate pronounced localized loading over the acromion, lateral clavicular region, and lateral cervical soft tissues during side-lying, thereby concentrating pressure on anatomically vulnerable areas. With increasing luffa content, all three pressure-related indicators exhibited a characteristic trend of an initial reduction followed by a slight rebound. Specifically, the maximum pressure decreased from 5.57 kPa to 4.72 kPa and then increased to 5.31 kPa; the average pressure declined from 2.64 kPa to 2.22 kPa before rising to 2.53 kPa; and the pressure index decreased from 4.71 kPa to 4.07 kPa and subsequently increased to 4.50 kPa. Notably, the L-P4% pillow achieved the lowest values across all parameters, demonstrating superior capability in alleviating localized loading within critical lateral pressure zones, particularly over the acromion and lateral cervical musculature. In the lateral posture, the soft-tissue layers overlying the acromion and lateral cervical muscles are relatively thin, rendering these regions especially susceptible to elevated localized pressure. When such pressure exceeds the capillary-closure threshold of approximately 32 mmHg (≈4.26 kPa), microcirculatory perfusion may be markedly compromised, potentially resulting in tissue ischemia and discomfort [50]. The L-P4% formulation demonstrated superior performance compared to other loadings, effectively reducing the area subjected to such critical pressure. Specifically, L-P4% resulted in the lowest maximum pressure, average pressure, and pressure index among all tested formulations (L-P0% to L-P8%), thereby promoting better perfusion and minimizing discomfort risk. This contrasts with L-P6% and L-P8%, which showed less effective pressure redistribution. Its maximum pressure approached the safety threshold and remained markedly lower than that of the unmodified L-P0% group. Such reductions are expected to better preserve microvascular perfusion in the shoulder–neck region during lateral sleeping and to mitigate numbness or discomfort associated with prolonged compression. Collectively, these findings highlight the superior lateral-support capability and physiological comfort afforded by the L-P4% formulation.

As shown in Figure 6g,h and summarized in Table S11 (Supplementary Materials), the contact area of the luffa-modified PU pillows under the lateral sleeping posture exhibited a characteristic trend of increasing initially and then decreasing with increasing luffa content. Compared with the unmodified L-P0% pillow, both L-P2% and L-P4% showed increased total contact area, with L-P4% achieving the highest value, increasing from 469.38 cm^2^ to 474.12 cm^2^ (an overall increase of 10.01%). In contrast, the total contact areas of L-P6% and L-P8% were lower than that of L-P0%. With respect to the pressure-stratified contact areas (s1–s4), the L-P4% pillow exhibited the largest area in s1 (low-threshold pressure region), reaching 153.50 cm^2^, indicating an enhanced capacity to expand low-pressure load-bearing zones. Conversely, in both s3 (higher-threshold pressure) and s4 (high-threshold pressure) regions, L-P4% showed the smallest contact areas, demonstrating its effectiveness in limiting high-pressure concentrations that may exceed soft-tissue tolerance. From a tissue biomechanics perspective, lateral sleeping tends to concentrate pressure on regions with relatively thin soft-tissue coverage, such as the acromion, lateral clavicular area, and lateral cervical musculature, where excessive localized loading may exceed the capillary closure pressure of approximately 32 mmHg (≈4.26 kPa), thereby impairing microvascular perfusion and increasing the risk of tissue ischemia [50]. By increasing the total contact area, enlarging the low-pressure s1 region, and markedly reducing the high-pressure s3–s4 zones, the L-P4% formulation promoted a more uniform load redistribution within pressure levels that are more tolerable to soft tissues. Such a reduction in high-pressure points exceeding the capillary-perfusion threshold is conducive to maintaining microvascular blood flow and metabolic homeostasis in the shoulder–neck region during lateral sleeping. Considering the quantitative outcomes, the L-P4% formulation exhibited the most favorable overall performance, characterized by the largest total contact area, the most substantial expansion of low-pressure regions, and the greatest reduction in high-pressure zones. Collectively, these results indicate that a luffa incorporation level of 4% provides the most effective load dispersion and localized pressure alleviation under the lateral sleeping posture.

#### 3.5.2. Influence of Pillow Height on Pressure-Relief Performance

**Figure 7:**
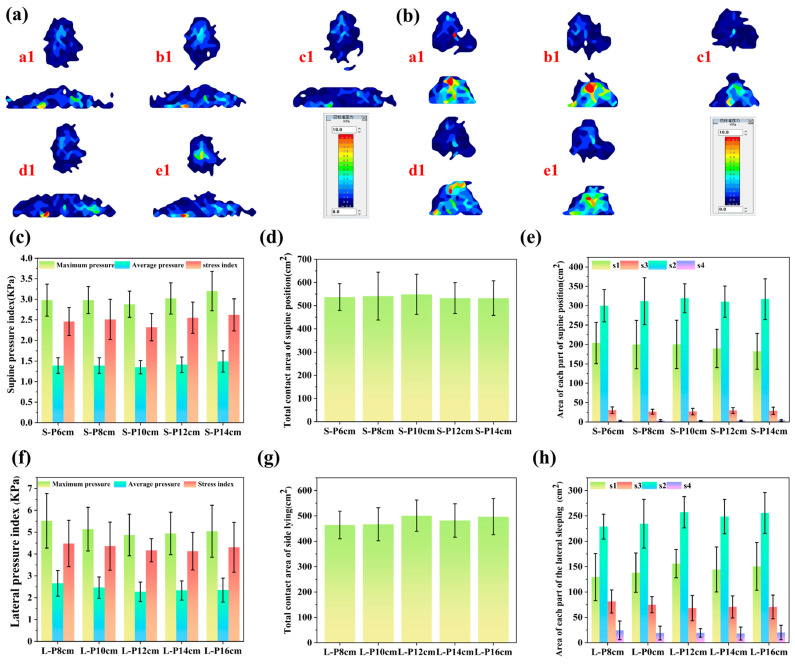
(**a**) Body pressure distribution at the human–occipital interface for (**a1**) S-P6 cm, (**b1**) S-P8 cm, (**c1**) S-P10 cm, (**d1**) S-P12 cm, (**e1**) S-P14 cm (supine position). (**b**) Body pressure distribution at the human–occipital interface for (**a1**) L-P8 cm, (**b1**) L-P10 cm, (**c1**) L-P12 cm, (**d1**) L-P14 cm, and (**e1**) L-P16 cm (lateral position). (**c**) Pressure indicators for different pillow heights (supine position). (**d**) Contact areas of different pillow heights in supine position: total contact area. (**e**) Contact areas of different pillow heights in supine position: s1: Contact area within the pressure range below 0.67 kPa, s2: Contact area within the pressure range of 0.67–4 kPa, s3: Contact area within the pressure range of 4–9.33 kPa and s4: Contact area within the pressure range of 4–9.33 kPa. (**f**) Pressure indicators for different pillow heights (lateral position). (**g**) Contact areas of different pillow heights in the lateral position: total contact area. (**h**) Contact areas of different pillow heights in the lateral position: s1: Contact area within the pressure range below 0.67 kPa, s2: Contact area within the pressure range of 0.67–4 kPa, s3: Contact area within the pressure range of 4–9.33 kPa and s4: Contact area within the pressure range of 4–9.33 kPa.

As shown in Figure 7a, the body–pillow interface pressure distribution of the same participant in the supine position was assessed using 4% luffa-modified PU pillows with different heights (6, 8, 10, 12, and 14 cm). The pressure maps indicate that, under the supine posture, the deep-red high-pressure regions exhibited a characteristic trend of decreasing initially and then increasing as pillow height increased. For the S-P14 cm pillow, the pressure distribution across the head and shoulder regions became noticeably non-uniform, with pronounced variations in color intensity and evident stress concentration. Similar localized high-pressure zones were also observed in the shoulder region for the S-P8 cm and S-P14 cm pillows, suggesting suboptimal load redistribution at these heights. In contrast, the S-P10 cm pillow produced a substantially more uniform pressure distribution, with improved load sharing between the head and shoulder regions. The narrower range of color variation in the pressure map reflects reduced fluctuations in localized pressure, thereby effectively alleviating interface loading transmitted to the user. Collectively, these results indicate that, for the supine sleeping posture, the 4% luffa-modified PU pillow achieves its most favorable pressure-relief performance at a height of 10 cm, providing the greatest comfort enhancement under this condition.

As shown in Figure 7c and Table S12 (Supplementary Materials), the pressure-related parameters of the luffa-modified PU pillows in the supine posture exhibited a characteristic decrease–increase pattern with increasing pillow height. When the height increased from 8 cm to 10 cm, the maximum pressure decreased from 2.98 kPa to 2.88 kPa, the average pressure declined from 1.39 kPa to 1.35 kPa, and the pressure index dropped from 2.46 kPa to 2.32 kPa, collectively indicating the most favorable pressure-relief performance. However, when the height was further increased to 14 cm, all three indicators increased to 3.20 kPa, 1.49 kPa, and 2.62 kPa, respectively, suggesting that an excessively elevated pillow height promotes renewed concentration of localized loading, particularly in the occipital region. From a biomechanical perspective, the supine posture requires the pillow to provide coordinated support for the occiput, the soft tissues near the superior nuchal line, and the physiological cervical lordosis. Insufficient pillow height limits the ability to maintain a neutral cervical alignment, thereby increasing occipital loading. Conversely, excessive height induces passive cervical flexion and anterior translation of the head, generating counter-directional compressive stresses on the occiput and upper cervical segments and reducing the uniformity of pressure distribution. A comparative assessment of pillow heights revealed that the 10 cm configuration (S-P10 cm) provided the optimal pressure relief in the supine position. While all tested heights maintained maximum pressure below the capillary closure threshold (4.26 kPa),the S-P10 cm pillow exhibited the lowest maximum pressure (2.88 kPa), average pressure (1.35 kPa), and pressure index (2.32 kPa) compared to the 8 cm, 12 cm, and 14 cm heights. This indicates that 10 cm best supports neutral cervical alignment and promotes homogeneous load distribution. These results indicate that a pillow height of 10 cm most effectively maintains the cervical spine in a neutral anatomical alignment and facilitates a more homogeneous redistribution of pressure across the occipital contact region, thereby supporting adequate perfusion of the occipital and upper cervical tissues. Considering both biomechanical requirements and perfusion-related safety criteria, 10 cm can be regarded as the most appropriate pillow height for the supine sleeping posture. By contrast, heights beyond this level (e.g., 14 cm) may disrupt cervical mechanical equilibrium and increase localized occipital loading, ultimately compromising sleep comfort. Accordingly, pillow-height selection should be incorporated as a key ergonomic parameter in pillow design to optimize comfort while maintaining physiological support in the supine position.

As shown in Figure 7d,e, and Table S13 (Supplementary Materials), the contact area of the luffa-modified PU pillows in the supine posture exhibited a characteristic trend of increasing initially and then decreasing with rising pillow height. Among all configurations, the 10 cm pillow (S-P10 cm) showed the largest total contact area, indicating that it provides the broadest load-bearing region at the body–pillow interface. Within the pressure-stratified regions (s1–s4), S-P10 cm exhibited the largest contact areas in the low-pressure ranges (<0.67 kPa and 0.67–4 kPa), suggesting that this height effectively redistributes a greater proportion of the interface load into low-threshold zones and thereby alleviates localized stress concentrations in the occipital and upper cervical soft tissues. In contrast, its contact area in the 4–9.33 kPa range (s3) was comparatively smaller, and in the >9.33 kPa high-threshold range (s4), it was the smallest among all pillow heights, indicating a marked reduction in high-pressure regions and, consequently, the most uniform overall pressure distribution at this configuration. Biomechanically, the supine posture requires coordinated support of the occiput, the musculature near the superior nuchal line, and the physiological cervical lordosis; thus, an appropriate pillow height must not be excessively low, which can concentrate pressure on the occipital region, or excessively high, which may induce passive cervical flexion and elevate interface pressure. By substantially enlarging the low-pressure regions (s1–s2) and reducing the contact area associated with higher-pressure zones (s3–s4), the S-P10 cm configuration promotes a more homogeneous pressure distribution. Furthermore, the maximum pressure at this height remained well below the capillary closure threshold, thereby supporting adequate microvascular perfusion in the occipital and posterior cervical soft tissues. In summary, the 10 cm pillow height demonstrated the most favorable supine-posture performance, characterized by optimal utilization of the supporting contact area, pronounced expansion of low-pressure regions, effective attenuation of high-pressure zones, and maintenance of tissue perfusion within physiologically safe limits. Collectively, these attributes indicate that a pillow height of 10 cm represents the most ergonomically appropriate and medically safe configuration for supine sleeping.

As shown in Figure 7b, the body–pillow interface pressure distribution of the same participant in the lateral sleeping posture varied markedly among luffa-modified PU pillows with different heights. The evaluated heights under the lateral condition were 8, 10, 12, 14, and 16 cm. Compared with the supine posture, the lateral pressure maps exhibited greater non-uniformity, which is primarily attributable to the reduced body–pillow contact area in side-lying and the consequent concentration of load within a smaller support region. Pronounced red areas were observed for the L-P8 cm and L-P10 cm pillows, indicating evident stress concentration. With increasing pillow height, the L-P12 cm configuration produced a more uniform pressure distribution, reflecting improved load redistribution at the body–pillow interface. However, further increases in height (L-P14 cm and L-P16 cm) were associated with greater variation in color intensity, suggesting the re-emergence of localized high-pressure zones relative to the more homogeneous distribution achieved by the L-P12 cm pillow. Pillow height is a critical determinant of sleep quality and musculoskeletal health. In the supine posture, excessive pillow height can induce anterior cervical flexion and, over time, may contribute to cervical spine disorders; conversely, an overly low pillow provides insufficient cervical support, potentially leading to cervical muscle fatigue and discomfort [51]. Under the lateral posture, pillow height should adequately fill the gap between the head and the mattress to maintain near-horizontal alignment of the head and spinal column. If the pillow is excessively high or too low, the cervical spine and upper thoracic segments deviate from neutral alignment, generating torsional loading. Such malalignment can impose sustained tension on the cervical and shoulder musculature and, with prolonged use, may lead to neck and shoulder pain, headaches, or discomfort extending to the thoracic and lumbar regions [10].

As shown in Figure 7f and Table S14, the pressure performance of the 4% luffa-modified polyurethane foam pillow in the lateral posture exhibited moderate yet biomechanically meaningful differences with changes in pillow height. The maximum pressure did not follow a clear linear trend; among the tested conditions, L-P16 cm produced the highest maximum pressure (5.04 kPa), whereas L-P12 cm yielded the lowest value (4.87 kPa), suggesting that excessively high pillow heights may increase localized loading, particularly over the acromial region. The average pressure showed a slight overall decrease with increasing pillow height, with L-P8 cm exhibiting the highest average pressure (2.66 kPa) and L-P12 cm the lowest (2.27 kPa), indicating that a moderate increase in pillow height can improve geometric compatibility between the head and shoulder in the lateral posture and facilitate more effective redistribution of localized loads. The pressure index displayed a similarly differentiated pattern, reaching its highest value for L-P8 cm (4.48 kPa) and its lowest for L-P14 cm (4.13 kPa), reflecting height-dependent differences in pressure-distribution uniformity. Biomechanically, a pillow used in the lateral posture must provide coordinated support to the lateral occipital region, cervical musculature, and acromial area. When pillow height is insufficient, the head tends to sink downward, promoting concentrated loading on the acromion and lateral clavicular soft tissues; conversely, an excessively high pillow can induce excessive lateral bending of the cervical spine, increasing tensile and compressive stresses in the lateral cervical musculature and contributing to renewed pressure concentration. Consistent with these biomechanical considerations, the quantitative results indicate that the L-P12 cm configuration provided the most favorable overall performance across the key metrics, achieving the lowest maximum pressure (4.87 kPa) and the lowest average pressure (2.27 kPa), together with the most advantageous pressure-index level among the tested heights, indicating improved pressure dispersion and interface uniformity. This height supported a near-neutral cervical alignment during lateral sleeping and promoted a more homogeneous redistribution of pressure within soft tissues capable of tolerating compression. Notably, although the maximum pressures observed under all pillow heights were above the capillary closure threshold of 32 mmHg (≈4.26 kPa), the L-P12 cm pillow demonstrated the strongest pressure-dispersion capability and most effectively reduced the extent of high-pressure regions approaching this threshold. Collectively, these findings suggest that a pillow height of 12 cm provides superior physiological comfort and improved perfusion safety under the lateral sleeping posture.

As shown in Figure 7g,h, and Table S15 (Supplementary Materials), the contact area of the luffa-modified PU pillows in the lateral posture exhibited a characteristic increase–decrease trend with increasing pillow height, with the L-P12 cm configuration achieving the largest total contact area and thus providing the most complete conformity to the soft tissues over the acromion, lateral clavicular region, and lateral cervical musculature. Within the pressure-graded regions, L-P12 cm also showed the largest contact areas in s1 (low-threshold) and s2 (lower-threshold), indicating its ability to substantially expand low-pressure support zones and reduce localized loading on anatomically vulnerable regions. By contrast, the L-P10 cm configuration exhibited the smallest contact area in s3 (higher-threshold) and remained comparatively low in s4 (high-threshold), suggesting that moderate pillow heights are more effective in suppressing the formation of high-pressure regions and in generating a more favorable pressure-distribution profile. From a biomechanical perspective, the lateral sleeping posture requires the pillow to provide coordinated support to the lateral occiput, cervical musculature, and acromial region; insufficient pillow height allows the head to sink, thereby concentrating load on the acromion and adjacent soft tissues, whereas excessively high pillows induce excessive lateral bending of the cervical spine, increasing passive stretching of the scalene and sternocleidomastoid muscles and elevating soft-tissue compression, which can ultimately reduce total contact area and enlarge high-pressure zones [52]. Analysis of the contact areas and pressure distribution across different pillow heights identified 12 cm (L-P12 cm) as optimal for the lateral posture. The L-P12 cm pillow achieved the largest total contact area and the most significant expansion of low-pressure zones (s1, s2), concurrently exhibiting the smallest contact areas in the high-pressure regions (s3, s4) compared to heights of 8 cm, 10 cm, 14 cm, and 16 cm. This effective load redistribution contrasts with the poorer performance of excessively low (e.g., 8 cm) or high (e.g., 16 cm) pillows, ensuring physiological safety and comfort. In contrast, excessively high or low pillow heights reduce the total contact area, limit the expansion of low- and medium-pressure zones, and enlarge high-pressure regions, thereby weakening pressure-relief capacity and reducing comfort in the lateral posture. Considering contact-area utilization, pressure-redistribution efficiency, and perfusion-related physiological safety, a pillow height of 12 cm best satisfies the biomechanical requirements of lateral sleeping and is therefore identified as the optimal configuration for side-lying support under the conditions of this study.

## 4. Conclusions

The incorporation of 4% luffa into polyurethane foam resulted in significant improvements in its cellular architecture, which, in turn, enhanced both the tensile properties and overall durability of the foam. This formulation also notably improved the moisture absorption and desorption performance, with increases of 19.4% in moisture uptake and 22.6% in moisture release. These enhancements are attributed to the increased porosity and the interconnected pore structure, which create additional pathways for moisture transport, facilitating more efficient moisture desorption. The improvements in moisture management not only contribute to superior functional performance but also enhance the service life of the foam, making it more effective and durable in practical applications. In the context of pillow applications, the 4% luffa foam demonstrated exceptional pressure relief and comfort in both supine and lateral sleeping postures, effectively reducing cervical muscle strain and improving local blood perfusion. The optimal pressure-relief effect was achieved with a pillow height of 10 cm for the supine posture and 12 cm for the lateral posture, promoting better sleep health and contributing to an improved overall quality of life.

## Data Availability

The original contributions presented in this study are included in the article/Supplementary Materials. Further inquiries can be directed to the corresponding author(s).

## Supplementary Materials

The following supporting information can be downloaded at: https://www.mdpi.com/article/10.3390/polym18030320/s1, Table S1: Bubble diameter of loofah-modified polyurethane foam; Table S2: Emulsion time, gel time and surface drying time of loofah-modified polyurethane foam; Table S3: CHysteresis loss rate, compression set coefficient and indentation hardness; Table S4: Tensile strength, elongation at break and tear strength; Table S5: Density and porosity; Table S6: Root Mean Square Error and Pearson’s Correlation Coefficient of Moisture Absorption Experiment and Moisture Absorption Simulation Experiment; Table S7: Root mean square error and Pearson’s correlation coefficient between experimental and simulated values; Table S8: Pressure indicator test results (supine position); Table S9: Contact area of sleeping pillows with different loofah additions in supine position; Table S10: Stress indicator test results (lateral position); Table S11: Contact area of sleeping pillows with different loofah additions in the lateral position; Table S12: Pressure indicator test results (supine position); Table S13: Contact area of different pillow heights in supine position; Table S14: Stress indicator test results (lateral position); Table S15: Contact area of different pillow heights in lateral position.
