# Supplementary material for: Bio-Inspired Microstructural Engineering of Polyurethane Foams with Luffa Fibers for Synergistic Optimization of Ergonomic Support and Hygrothermal Comfort
Source: Polymers (Basel). 2026 Jan 25;18(3):320. doi: 10.3390/polym18030320 (PMC12899512; doi:10.3390/polym18030320)
Supplement: Supplementary file 1 [file polymers-18-00320-s001.zip › polymers-4072652-supplementary.pdf]

Supplementary Materials

Table S1. Bubble diameter of loofah-modified polyurethane foam

Foam sample	The addition amount of loofah sponge(%)	Cell diameter (μm)
L-F0%	0	652±285.1
L-F2%	2	585±250.9
L-F4%	4	564±191.6
L-F6%	6	545±217.5
L-F8%	8	634±244.6

Table S2. Emulsion time, gel time and surface drying time of loofah-modified polyurethane foam

Sample	Characteristic reflection time		
	Emulsion time(s)	gel time (s)	surface drying time(s)
L-F0%	24.06±2.48	135.49±7.14	185.21±6.96
L-F2%	24.64±1.21	163.02±8.16	227.90±6.80
L-F4%	27.35±1.58	174.48±6.95	248.65±6.20
L-F6%	28.18±4.98	204.68±9.38	275.49±6.61
L-F8%	30.17±4.50	207.41±10.89	303.72±2.64

Table S3. CHysteresis loss rate, compression set coefficient and indentation hardness

Sample	CHysteresis loss rate (Af) （%）	compression set coefficient (Sf)	HB (25%/30s) （N）	HB (40%/30s) （N）	HB (65%/30s) （N）
L-F0%	26.3±0.12 b	2.70±0.00 d	12.59±0.01 c	19.24±0.02 d	34.01±0.07 e
L-F2%	25.6±0.12 c	2.70±0.01 d	15.94±0.02 a	24.12±0.02 a	42.96±0.01 a

L-F4%	26.29±0.11 b	2.81±0.01 c	14.37±0.02 b	22.00±0.02b	40.32±0.04 b
L-F6%	26.64±0.20 a	2.98±0.01 b	12.36±0.05 d	19.47±0.06 c	37.07±0.01 c
L-F8%	26.76±0.08 a	3.13±0.01 a	11.66±0.01 e	18.43±0.01 e	36.33±0.00 d
P	0.000	0.000	0.000	0.000	0.000

Table S4. Tensile strength, elongation at break and tear strength

Sample	Tensile strength（KPa）	Tensile elongation（%）	Tear strength（KPa）
L-F0%	42.39±0.90	112	7.0±0.35
L-F2%	44.67±1.55	120	7.8±0.40
L-F4%	45.73±0.80	129	7.1±0.18
L-F6%	44.39±2.97	125	6.9±0.26
L-F8%	39.05±1.89	100	6.1±0.15

Table S5. Density and porosity

Foam sample	The amount of silk sponge fibers added(%)	Density (kg m3)	porosity (%)
L-F0%	0	51.86±3.81	89.87±0.02
L-F2%	2	53.05±2.16	91.71±0.01
L-F4%	4	55.72±2.87	92.75±0.04
L-F6%	6	57.44±3.25	94.19±0.02
L-F8%	8	57.26±2.60	92.27±0.02

Table S6. Root Mean Square Error and Pearson's Correlation Coefficient of Moisture Absorption Experiment and Moisture Absorption Simulation Experiment

Type of material	Root Mean Square Error (RMSE)	Pearson correlation coefficient r

L-F0%	0.044	0.999
L-F2%	0.067	0.997
L-F4%	0.075	0.997
L-F6%	0.054	0.999
L-F8%	0.064	0.998

Table S7. Root mean square error and Pearson's correlation coefficient between experimental and simulated values

Type of material	Root Mean Square Error (RMSE)	Pearson correlation coefficient r
L-F0%	0.018	0.999
L-F2%	0.017	0.999
L-F4%	0.015	0.999
L-F6%	0.011	0.999
L-F8%	0.013	0.998

Table S8. Pressure indicator test results (supine position)

Sample	Maximum pressure（KPa）	Average pressure（KPa）	Stress Index（KPa）
L-P0%	3.21±0.49 ^{a}	1.50±0.27 ^{a}	2.64±0.41 ^{a}
L-P2%	3.12±0.28 ^{ab}	1.45±0.14 ^{ab}	2.63±0.26 ^{a}
L-P4%	2.81±0.50 ^{b}	1.29±0.23 ^{b}	2.43±0.40 ^{a}
L-P6%	3. 10±0.36^{ab}	1.42±0.16 ^{ab}	2.68±0.41 ^{a}
L-P8%	3.07±0.40 ^{ab}	1.41 ±0.22 ^{ab}	2.65±0.21 ^{a}
p	0.235	0.197	0.473

Table S9. Contact area of sleeping pillows with different loofah additions in supine position

Sample	Total contact area （cm2）	S1 （cm2）	S2 （cm2）	S3 （cm2）	S4 （cm2）
L-P0%	520.88±76.88 a	181.00±44.79 a	307.32±49.01 a	28.00±10.20 a	4.56±2.39 a
L-P2%	515.27±60.76 a	182.49±33.28 a	302.22±51.40 a	26.95±9.18 a	3.62±2.14 a
L-P4%	551.85±75.68 a	216.44±63.86 a	305.82±50.37 a	26.44±8.05 a	3. 16±2.97 a
L-P6%	536.86±83.63 a	193.55±30.75 a	310.55±61.16 a	29.00±5.85 a	3.75±2.19 a
L-P8%	534.12±67.00 a	192.51±46.36 a	309.05±44.73 a	29.30±11.08 a	3.26±1.84 a
P	0.784	0.376	0.996	0.932	0.682

Table S10. Stress indicator test results (lateral position)

Sample	Maximum pressure（KPa）	Average pressure（KPa）	Stress Index（KPa）
L-P0%	5.57±1.66 a	2.64±0.81 a	4.71±1.59 a
L-P2%	5.44±1.00 a	2.58±0.47 a	4.67±0.97 a
L-P4%	4.72±1.06 a	2.22±0.47 a	4.07±0.92 a
L-P6%	5.46±1.25 a	2.60±0.61 a	4.67±1.03 a
L-P8%	5.31±1.02 a	2.53±0.45 a	4.50±1.03 a
P	0.507	0.451	0.678

Table S11. Contact area of sleeping pillows with different loofah additions in the lateral position

Sample	Total contact area （cm2）	S1 （cm2）	S2 (cm2)	S3 (cm2)	S4 (cm2)
L-P0%	469.38±75.06 a	130.61±46.03 a	245.05±36.72 a	69.65±23.28 a	24.07±18.58 a
L-P2%	473.52±57.82 a	136.14±34.67 a	233.01±29.97 a	79.91±19.77 a	24.47±15.59 a

L-P4%	474. 12±48.07 a	153.50±29.43 a	238.54±34.49 a	64.91±15.60 a	17.17±16.17 a
L-P6%	469. 18±77.17 a	144.82±40.56 a	221. 18±39.93 a	77.93±30.67 a	25.25±17.75 a
L-P8%	464.09±42.18 a	128.95±27.51 a	236.93±23.92 a	73.25±16.45 a	24.96±17.60 a
P	0.995	0.466	0.547	0.492	0.785

Table S12. Pressure indicator test results (supine position)

Sample	Maximum pressure（KPa）	Average pressure（KPa）	Stress Index（KPa）
S-P6cm	2.98±0.39 a	1.39±0.19 a	2.46±0.34 a
S-P8cm	2.98±0.33 a	1.39±0.19 a	2.51±0.49 a
S-P10cm	2.88±0.32 a	1.35±0.16 a	2.32±0.33 a
S-P12cm	3.02±0.38 a	1.41±0.19 a	2.55±0.38 a
S-P14cm	3.20±0.48 a	1.49 ±0.26 a	2.62±0.39 a
P	0.361	0.529	0.422

Table S13. Contact area of different pillow heights in supine position

Sample	Total contact area（cm2）	S1（cm2）	S2（cm2）	S3（cm2）	S4（cm2）
S-P 6cm	541.40±103.16 a	199.67±62.28 a	311.54±60.42 a	26.64±6.22 a	3.55±2.23 ab
S-P 8cm	541.40±103.16 a	199.67±62.28 a	311.54±60.42 a	26.64±6.22 a	3.55±2.23 ab
S-P10cm	548.73±87.03 a	200.03±62.35 a	319.22±37.39 a	26.95±8.50 a	2.53±1.25 b
S-P12cm	532.63±66.71 a	189.54±49.29 a	310.36±40.26 a	29.66±7.42 a	3.07±1.72 ab

S-P14cm	532.45±74.63 [a]	182.49±46.11 [a]	316.87±52.65 [a]	28.76±9.58 [a]	4.34±2.27 [a]
P	0.985	0.871	0.873	0.724	0.135

Table S14. Stress indicator test results (lateral position)

Sample	Maximum pressure（KPa）	Average pressure（KPa）	Stress Index（KPa）
L-P 8cm	5.52±1.25 [a]	2.66±0.59 [a]	4.48±1.06 [a]
L-P10cm	5.14±1.00 [a]	2.46±0.49 [a]	4.36±1.10 [a]
L-P12cm	4.87±0.95 [a]	2.27±0.45 [a]	4.17±0.53 [a]
L-P14cm	4.94±0.97 [a]	2.33±0.44 [a]	4.13±0.86 [a]
L-P16cm	5.04±1.19 [a]	2.35±0.55 [a]	4.31±1.14 [a]
P	0.691	0.449	0.931

Table S15. Contact area of different pillow heights in lateral position

Sample	Total contact area（cm2）	S1（cm2）	S2（cm2）	S3（cm2）	S4（cm2）
L-P 8cm	464.09±54.48 [a]	129.50±46.51 [a]	228.79±24.60 [a]	81.39±22.58 [a]	24.42±18.42 [a]
L-P10cm	466.98±65.22 [a]	138.18±38.67 [a]	234.58±47.99 [a]	75.06±16.01 [a]	19.17±13.60 [a]
L-P12cm	500.98±61.99 [a]	155.90±27.84 [a]	257.18±30.81 [a]	68.37±24.93 [a]	19.53±8.57 [a]
L-P14cm	481.81±65.95 [a]	144.42±44.30 [a]	248.60±33.91 [a]	70.63±21.84 [a]	18.15±12.96 [a]
L-P16cm	497.00±71.24 [a]	150.48±46.96 [a]	255.74±40.30 [a]	70.54±23.35 [a]	20.26±14.35 [a]
P	0.609	0.690	0.312	0.661	0.861
